# The Effects of Heat Exposure During Intermittent Exercise on Physical and Cognitive Performance Among Team Sport Athletes

**DOI:** 10.1177/0031512520966522

**Published:** 2020-10-20

**Authors:** Kate Donnan, Emily L. Williams, Nicholas Stanger

**Affiliations:** 1 Carnegie School of Sport, Leeds Beckett University, Leeds, United Kingdom

**Keywords:** affect, catecholamines, cognitive function, core temperature, power output

## Abstract

This study investigated the effects of heat exposure on physical and cognitive performance during an intermittent exercise protocol so as to reflect the incremental fatigue experienced during team sports. Twelve well-trained male team sport players completed an 80-minute cycling intermittent sprint protocol (CISP), alongside computerized vigilance and congruent (i.e., simple) and incongruent (i.e., complex) Stroop tasks of cognitive functioning, in two counterbalanced temperature conditions; hot (32°C[50%rh]) and control (18°C[50%rh]). Incongruent Stroop accuracy declined over time (*p* = .002), specifically in the second (*M^diff^ =* –3.75, *SD* = 0.90%, *p* = .009) and third (*M^diff^ *= –4.58, *SD* = 1.22%, *p* = .019) quarters compared to the first quarter of the CISP; but there were no differences between temperature conditions. Congruent Stroop reaction time (RT) was quicker in the second quarter of exercise in the hot condition (*M* = 561.99, *SD* = 112.93 ms) compared to the control condition (*M*=617.80, *SD* = 139.71 ms; *p* = .022), but no differences were found for congruent Stroop accuracy nor vigilance measures. Additionally, peak power output was lower during the third quarter of the CISP in the hot condition (*M* = 861.31, *SD* = 105.20 W) compared to the control condition (*M* = 900.68, *SD* = 114.84 W; *p* < .001). Plasma normetanephrine and metanephrine concentrations increased from pre- to post-CISP (*M^diff^ =* +616.90, *SD* = 306.99, *p* < .001; and *M^diff^ *= +151.23, *SD* = 130.32, *p* = .002, respectively), with a marginal interaction suggesting a higher normetanephrine increase from pre- to post-CISP in the hot versus the control condition (*p* = .070). Our findings suggest that accuracy for more complex decisions suffered during prolonged high-intensity intermittent exercise, perhaps due to exercise-induced catecholamine increases. Athletes may have also reduced physical effort under increased heat exposure, indicating how cognitive performance may be sustained in physically demanding environments.

## Introduction

In team sports such as soccer, elite-level players often cover distances of 10 km in a single match, running at average intensities that are close to their anaerobic threshold, with numerous intermittent explosive bursts of activity (e.g. sprinting, jumping) (Stølen et al., 2005). However, effective performance is dependent upon a myriad of factors requiring simultaneous performance of a range of cognitive and perceptual skills (Williams, 2000). For example, prior to receiving the ball, a soccer player must show spatial awareness of vacant space and/or player positions before deciding where or when to play the ball. In such situations, players simultaneously apply vigilance, selective attention and working-memory of player positions under both time restrictions and physical exertion(Heppe et al., 2016). Research has revealed that most goals in soccer are conceded in the last 15-minutes of matches, suggesting that physical fatigue typically experienced in the latter stages of match-play may increase the likelihood of mistakes in cognitive skills (Alberti et al., 2013). High-intensity work has been shown to have been reduced across all positions in the last 15-minutes of match-play (Bradley et al., 2009), suggesting that the increase in goals scored late in a game could be due to players capitalizing on mistakes, rather than to an increased intensity of the offense. Progressive fatigue observed from prolonged exercise, such as during team sport performance, has been attributed to diminished muscle glycogen stores, hyperthermia, increased loss of body fluids, and altered synthesis and metabolism of neurotransmitters (Meeusen et al., 2006; Mohr et al., 2012).

In addition to match-play induced fatigue, many team sports particularly at the elite level, are performed in hot and humid conditions in which relative humidity (rh) can exceed 30°C and 70%rh. For instance, during the 2014 Soccer World Cup held in Brazil at least 25% of matches were played under conditions classified as ‘high risk’ of heat illness/injury (Nassis et al., 2015), and major competitions continue to be scheduled in equatorial or Middle-Eastern regions (e.g., 2022 Qatar Soccer World Cup). In such extreme environments, during prolonged high-intensity intermittent exercise, core temperature, and individual sweat responses are often significantly elevated (Shirreffs et al., 2005), potentially leading to adverse consequences such as hyperthermia (Maughan, 2010). Both physical and cognitive performances may be hindered by these core temperature changes, perhaps influencing overall sport performance.

The central fatigue hypothesis has explained that, during prolonged exercise, the synthesis and activity levels of central catecholamines (e.g. dopamine, norepinephrine) and serotonin are altered, potentially limiting aspects of physical and cognitive performance (Davis & Bailey, 1997; Meeusen et al., 2006). It has also been suggested that catecholamine and serotonin activity play a role in heat tolerance, as catecholaminergic and serotoninergic projections are known to stimulate regions of the hypothalamus, otherwise known as the thermoregulatory center (Meeusen et al., 2006). When stress increases to a moderate level in response to acute moderate intensity exercise, brain catecholamine concentrations rise. This normally results in a norepinephrine induced increased firing of high-affinity α_2A_-adrenoreceptors, and an increased activation of high-affinity D1 dopaminergic receptors (Arnsten, 2000), leading to inhibited firing to non-preferred stimuli. This process reflects an improved ‘signal’ to ‘noise’ ratio that can result in optimized cognitive performance (McMorris & Hale, 2015). However, when experiencing high levels of stress, such as that induced by high-intensity exercise in the heat, norepinephrine and dopamine concentrations may rise to excessive levels, resulting in reduced neuronal firing and inducing additional activity of a secondary messenger, thereby dampening neural activity and weakening the ‘signal’ to ‘noise’ ratio in the prefrontal cortex (Arnsten, 2000; McMorris & Hale, 2015), associated with diminished cognitive accuracy.

Although, catecholamines have been shown to affect cognitive functioning, and to be influenced by exercise (e.g., McMorris & Hale, 2015) and acute environmental stress (e.g., Kishore et al., 2013), no research has yet investigated the simultaneous effects of heat during the physical exertion typically experienced in team sport on cognitive functioning and catecholamines. Therefore, there is a need to investigate the extent to which changes in central catecholamines account for performance maintenance and fatigue during a type of exercise that replicates the durations and intensities of heat exposure during match-play. To date, although research has demonstrated clear *physical* performance impairment under high-intensity intermittent exercise with heat exposure reflective of match-play in team sports (e.g., Aldous et al., 2016), it is still unclear what aspects of *cognitive* function are affected by these conditions.

Generally, it has been proposed that less complex cognitive functions, such as reaction time, are not as vulnerable to heat stress and may actually be improved via increased physiological arousal, even while higher-order cognition (i.e., executive functioning) may be jeopardized (Gaoua, 2010; Hancock & Vasmatzidis, 2003). However, debate persists with regard to the combined effects of exercise and heat exposure on cognitive function. Some researchers found cognitive decrements (McMorris et al., 2006), while others found no effects (Taylor et al., 2014), or even beneficial effects of prolonged exercise in the heat (Macleod et al., 2018). Some of these differences are likely to be due to the timing of the cognitive task employed, where most have investigated cognitive functions pre- and post-exercise only (e.g., Coull et al., 2015; Macleod et al., 2018; Taylor et al., 2014). A meta-analysis by Lambourne and Tomporowski (2010) observed that cognitive performance was typically impaired *during* exercise but was generally improved *following* exercise, reinforcing the need for greater consideration of the timing of the cognitive tasks employed. The transient hypofrontality hypothesis explains that during exercise, neural resources are diverted toward areas of the brain responsible for performing movement (i.e., the primary motor cortex) leaving fewer resources available for brain areas such as the prefrontal cortex, responsible for complex cognitive functioning (Dietrich, 2006). However, just moments after the cessation of exercise, neural activation has been shown to rapidly return to baseline (Dietrich, 2006). Therefore, differences in the timing of these cognitive tasks could be the basis for significant variances in past research results. Despite increased recent research on the cognitive effects of exercise-induced fatigue, there is still limited research that has investigated cognitive function *during* exercise, with some attempts to test these effects having relied exclusively on pre-, half-time and post-exercise cognitive assessments (e.g., Coull et al., 2015; MacLeod et al., 2018; Taylor et al., 2014) that do not fully reflect the simultaneous physical and cognitive demands of team sport.

In this study, we aimed to investigate the effects of heat exposure on both physical and cognitive performance during prolonged intermittent exercise reflective of match-play in team sport by comparing these effects in a hot (i.e., at 32°C) versus a temperate (control) temperature (i.e., at 18°C; neither hot nor cold) condition. We examined physical performance via power output, and cognitive performance via tests of both simple (i.e., numerical vigilance) and higher-order (i.e., interference control, selective attention) cognitive functioning. Moreover, we studied the potential biological mechanisms that might explain the potential effects of heat exposure during prolonged intermittent exercise on cognitive and physical performance by exploring potential roles of serotonin and metanephrines (i.e., indicators of norepinephrine, epinephrine, and dopamine), measured as the more stable extra-neuronal metabolites of catecholamine activity (Woods et al., 2017). We hypothesized that sprint power output would decline over time and that cognitive functioning would be impaired in response to exercise-induced fatigue, which would be exacerbated by increased heat stress. We expected metanephrines, particularly normetanephrine, to increase from pre- to post-exercise to a greater extent in the hot versus control condition.

## Method

### Participants

We recruited 12 well-trained male team sports players (*M* age = 21.4 years, *SD* = 3.3*;* SD = 3.6) from a university in the United Kingdom. Participants were classified as well-trained, where mean maximal oxygen uptake (VO_2max_) and body fat percentage values (*M* VO_2max_ = 53.0 mL·kg^−1^·min^−1^; *M* body fat percentage = 13.7%, *SD* = 2.4) aligned with previous research involving participants with similar characteristics (Leeder et al., 2014; Silvestre et al., 2006). Participants were recruited via email, word-of-mouth, from advertisements and via social media platforms. Participants competed in soccer (*n* = 6), rugby (*n* = 4), basketball (*n* = 1) and futsal (*n* = 1) and they averaged 11.7 years (*SD* = 4.2) playing experience in their respective sports. All participants regularly trained in their main sport at least twice-a-week and competed in at least one competitive match-a-week. We estimated this required sample size based on data from a similar study (McMorris et al., 2006) that examined the effects of heat and exercise on cognition and biochemical variables (i.e., adrenaline and noradrenaline) and found large to very large effects (Cohen’s *d* = .76 to 1.6). In a power analysis using G*Power 3.1.9.4 (Faul et al., 2007), we assumed a large effect size (i.e., Cohen’s *d* = 0.8; f = 0.4) with power of 1– β = .80 and statistical significance set at alpha = .05, and we determined a required sample size of nine participants. We recruited 12 participants to ensure full counterbalancing of the two temperature conditions in this study.

We asked participants to refrain from supplemental ergogenic aids for the duration of the study and to perform no strenuous exercise at temperatures >30°C for more than three months prior to the study, following procedures by Aldous et al. (2016). We also required participants to standardize their food intake by consuming the same foods on the morning of each trial, abstain from alcohol, cigarettes and caffeine for at least 24 hours before the experiment, refrain from strenuous exercise for at least 48 hours prior to the experiment, and maintain their regular sleep pattern for at least 72 hours before each trial, again following procedures used by Aldous et al. (2016). Participants completed food and sleep diaries prior to each trial in order to confirm their adherence to these experimental instructions. We instructed participants to arrive at least two hours post-prandial and to consume between 2 and 3 L of water in the 24 hours leading to testing. Following approval of this research protocol from the university research ethics committee, all participants who met these inclusion criteria provided their written informed consent before commencing this research.

### Research Design

This research design was a two-condition, within-subjects design in which all participants completed experimental high intensity exercise trials in a temperate control condition (18°C; 50% rh) and in an experimental hot (32°C; 50% rh) condition; participants undertook these trials in a randomized, counterbalanced order. Participants visited the laboratory on four occasions over a period of 28 days; two visits for familiarization, and two visits in which they conducted the high intensity exercise trials (i.e. the hot and control condition on separate visits) in an environmental chamber.

### Procedure

#### Familiarization Visits

During the first familiarization visit, after completing a medical pre-screening questionnaire, we recorded the participants’ body fat (%), body mass (kg) and height (cm), using a Bod Pod (Life Measurement Instruments) and Seca Stadiometer (Seca 220, Germany), respectively. Participants then completed a VO_2max_ test and, following a ∼15-minute recovery, they completed the Stroop and vigilance cognitive tests. Participants were then required to attend a second familiarization visit no less than three days, and no more than seven days, following their initial visit. During this, we familiarized participants with the exercise protocol by having them complete 20-minutes of the Cycling Intermittent Sprint Protocol (CISP) (Castle et al., 2006; Hayes et al., 2013) while they simultaneously performed the same cognitive tasks taken earlier (i.e., Stroop and vigilance tests).

#### Experimental Visits

Participants next completed the two main exercise trials in a randomized and counterbalanced order under the temperate control condition (*M* temperature = 18.0°C, *SD* = 1.0; *M* rh = 51.9%, *SD* = 3.5), and the hot condition (*M* temperature = 31.6°C, *SD* = 1.2; *M* rh = 49.3%, *SD* = 3.8). The interval between these two trials averaged five days (*SD* = 1). To minimise anticipatory effects, we informed participants before each trial that they would be performing the CISP in conditions between ∼15--35°C. The time of day that testing took place for each participant remained consistent across the two trials to control for circadian rhythm variations and their subsequent effects on core temperatureand power output (e.g., see Drust et al., 2005; Racinais et al., 2012). Hydration status was assessed prior to each trial via urine osmolality (Osmomat 030-D, Gonotec) and participants were only able to proceed with testing if their urine osmolality was <600 mOsm/l (e.g., Taylor et al., 2014). Ad libitum water intake was permitted throughout the CISP and during the half-time period, and total consumption (ml) was recorded for analysis. Power output, cognitive function and perceptual measures were each assessed on a per-quarter basis to make time inferences, where a quarter was defined as each 20-minute period (i.e., per 10-sprints).

### Assessment Measures

**VO_2_ max.** As noted above, we performed a VO_2max_ test during the initial familiarization visit, using a cycle ergometer (Lode Excalibur Sport 925900, Netherlands). To determine VO_2max_, we instructed participants to cycle at a self-selected pedal rate between 70--90 rpm. The VO_2max_ test began at 75 W, after which the work rate increased by 25 W.min^−1^ until participants could no longer maintain their cadence. The test was terminated if there was a greater than 10 rpm decline in cadence or through the participant’s report of exhaustion. We collected breath-by-breath pulmonary gas exchange throughout the incremental tests (Cortex, Metalyser 3B, Germany), and we averaged data across 10-s periods for analysis. VO_2max_ was identified as the highest 30-s mean value attained before the point of volitional exhaustion. We also recorded power output (W) from the final completed minute in order to calculate the active recovery stage prior to the CISP.

### CISP

**Figure 1.**
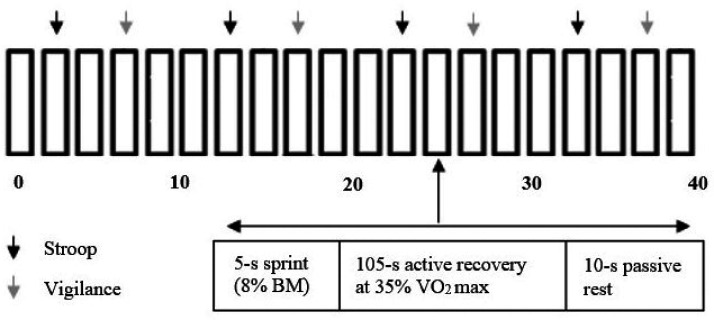
Modified Version of Hayes et al. (2013) Cycling Intermittent Sprint Protocol (CISP) Schematic, Representing One Half of the Extended CISP in This Research. Note: BM = body mass.

The exercise protocol that we used in the main high intensity exercise trials was the CISP (see Figure 1), a protocol that has been previously employed in several studies, aiming to replicate the physical effort demands of one half of a competitive team sport match (e.g., Castle et al., 2006; Hayes et al., 2013). This exercise was performed on a Lode Excalibur Sport cycling ergometer (925900, Netherlands). Each of the two trials consisted of a standardized 5-minute warm-up of cycling at 95 W at a cadence of 80 rpm, followed by two 30-second periods of passive rest, interspersed with 30-second cycling at 120 W (Hayes et al., 2013). The CISP typically consists of 20 blocks of 2-minute cycling periods, each comprised of a 5-second ‘all out’ maximal sprint against a resistance of 7.5% body mass (BM) beginning from a static position, a 105-second period of active recovery at 35% VO_2_ max and then a passive rest period of 10-seconds (Castle et al., 2006). With the Lode Excalibur Sport ergometer, we rounded sprint resistance to 8% BM. Throughout the period of active recovery, participants were required to maintain 70 rpm on the ergometer. In each trial, as the typical CISP replicates one half of the physical effort demands of team sport (e.g., Castle et al., 2006), participants performed an extended version of the CISP comprised of two 40-minute halves interspersed with a 15-minute “half-time” period. Thus, in each CISP trial in the present research, there was a total of 40 blocks of 2-minutes to simulate the demands of a typical team sport competitive match. The 15-minute half-time period included passive recovery in a temperate environment (∼18°C).

### Cognitive Measures

Participants were required to engage in Stroop and vigilance tasks at different time points throughout each CISP trial. These tasks were administered on E-Prime 3.0 software (Psychology Software Tools, Inc, Pittsburgh, USA). The Stroop and vigilance tests were each 60-seconds in length, and were first administered at rest, in a moderate environment (∼18˚C) before entering the environmental chamber, and then re-administered throughout the CISP. Both tests were presented on a computer screen that was positioned at eye level in front of the cycle ergometer. Participants were required to respond to the stimuli by using the response controls fixed to the handlebars of the ergometer.

#### Stroop

We used the Stroop test to measure information processing speed, interference control and selective attention (Adleman et al., 2002). Participants were presented with a series of words representing colors (e.g. red, blue, yellow, green) in either a ‘neutral’ colored font, or printed in ink colors of red, blue, yellow, or green, against a white background. We delivered congruent stimuli in which the color word was printed in the same ink color as the word (e.g., the word blue printed in blue ink) and incongruent stimuli in which the color word was printed in a different ink color (e.g., the word blue printed in red ink). The Stroop tasks were of 60-second length and involved 10 cases of each stimuli type (i.e., congruent, and incongruent) randomly presented. For each trial, these tasks were administered during the active recovery periods of the 2nd, 7th, 12th and 17th 2-minute cycling epochs of the half of the CISP (see Figure 1). To respond, participants were instructed to press the button that corresponded to the color of the font (ignoring what the word represented) as quickly as possible. We calculated participants’ scores in terms of their total accuracy (number of correct responses) and reaction time (ms) for both the congruent, and incongruent stimuli, and we then averaged these scores for each quarter of the CISP for later analysis. As per previous research employing the Stroop task (Malcolm et al., 2018), only reaction times of correct responses were used for analysis. Additionally, minimum (<100 ms) and maximum (>1500 ms) reaction times were employed to exclude any anticipatory or delayed responses (Malcolm et al., 2018), however no correct response recorded reached these values. The reliability of the Stroop has previously been assessed where low variability (CV <10%), and very good reproducibility (ICCs 0.71-.0.79, for congruent and incongruent stimuli, respectively) has been found (Cooper et al., 2015b; Strauss et al., 2005).

#### Vigilance

To measure participants’ vigilance, we used a slightly modified version of the numerical vigilance task used by Coull et al. (2015). This 60-second task involved 3-digit numbers that were flashed on the computer screen every 250 ms. Some numbers appeared twice in a row, at a duplication rate of 10%. Participants were instructed to respond to any duplicated set of digits by pressing the spacebar as quickly as possible. The vigilance task was administered to participants during each active recovery period of the 4th, 9th, 14th and 19th 2-minute cycling epochs during each half of the CISP (Figure 1). We calculated scores in terms of total accuracy (number of correct responses) and reaction time (ms), and we averaged these measures for each quarter of the CISP for later analysis.

### Physiological Measures

#### Peak Power Output (PPO)

Peak Power Output (W) was calculated as the highest power output recorded during each sprint of the CISP. We averaged this measure and analyzed it per quarter of the CISP due to doubling the length of the exercise protocol and to observe differences between specific time periods during match-play (i.e., beginning and end of each half).

#### Core Temperature (T_C_)

We measured T_C_ with a temperature sensor telemetry system (CorTemp®, HQinc, US), using a small pill that participants ingested ∼5-hours prior to exercise (Casa et al., 2007). We recorded T_C_ first pre-trial, following 15-minutes of seated rest, in order to allow resting values to stabilize. We then recorded T_C_ 1-minute into each 2-minute exercise block throughout the CISP. As per Hunt et al. (2017), we corrected sensor readings using the following equation: corrected temperature (°C) = 1.00375×sensor temperature (°C) − 0.205549, in order to help restrict systematic bias to within a+0.1˚C accuracy range. We averaged and analyzed this measure per quarter of the CISP.

#### Heart Rate (HR)

After participants sat passively for 15-minutes to allow resting values to stabilize, we first recorded their HR using a polar HR monitor (FT1, Polar, Finland). We then recorded HR 1-minute into each 2-minute exercise block throughout the CISP. We averaged and analyzed this measure per quarter of the CISP.

#### Metadrenalines and Serotonin Concentration

For measurement of plasma metadrenalines, we first collected three ml blood samples into EDTA tubes at rest (i.e., pre-CISP) from an indwelling venous catheter located in the arm. We then collected three ml blood samples again immediately post-CISP. These were centrifuged immediately (ALC; PK120R), and plasma was separated for analysis of plasma metadrenaline (MET) and normetanephrine (NMET) as trace indicators of plasma epinephrine and norepinephrine, respectively. These samples were frozen at –80°C until analysis by liquid chromatography–tandem mass spectrometry in the Integrated Laboratory Medicine Department, Freeman Hospital, Newcastle upon Tyne. Whole blood samples (2 ml) for serotonin concentration assay were also collected pre- and post-CISP into EDTA tubes and frozen immediately at –20°C. Following the cessation of exercise from each experimental session, the frozen blood samples were transported to an external laboratory on ice for analysis and analyzed by high-performance liquid chromatography by the Department of Specialist Laboratory Medicine, St James’s University Hospital, Leeds.

#### Perceptual Measures

During each trial, we obtained the participants’ ratings of perceived exertion (RPE) on the Borg Scale (Borg, 1982), and we recorded their reported thermal sensations at the end of each 10-minute block of the CISP. The Borg Scale was anchored from 6 (*no exertion*) to 20 (*maximal exertion*), and participants reported their thermal sensations on a Likert scale anchored from 0 (*unbearably cold*) to 8 (*unbearably hot*) (Toner et al., 1986). We also measured participants’ reports of affective valence once every 10-minutes of the CISP, where participants indicated their affective valence on the Feeling Scale (Hardy & Rejeski, 1989), anchored from –5 (*very bad*) through 0 (*neutral*) to 5 (*very good*). Previous research assessing the reliability of the Borg and Feeling Scale has found excellent agreement during exercise (ICC = 0.77, ICC = 0.83, respectively) within individuals across three sessions (Unick et al., 2015). Additionally, Toner et al.’s (1986) thermal sensation scale has been shown to be a valid measure of perceived heat stress, where correlations with large effect sizes (*r* = .72) have been shown between thermal sensation ratings and rectal temperature (Casa et al., 2007). Participants were asked to respond as to how they felt in that specific moment for all perceptual scales.

### Data Analysis

We completed statistical analyses using IBM SPSS statistics 24.0 (IBM Corporation, New York, United States). After data screening and checking for normality of the data distribution, we performed a series of two-way (temperature condition by time) repeated measures ANOVA’s to analyze condition differences on indicators of physical and cognitive performance, perceptual measures (e.g., RPE, as per Taylor et al., 2014) and most physiological variables (i.e., heart rate, core temperature, urine osmolality, metanephrines and serotonin). All pre-trial cognitive tests were first analyzed to ensure there were no differences at rest (*n* = 11) between conditions before analyzing quarter time periods^1^.

We used a one-way repeated measures ANOVA to analyze fluid intake across conditions. When significant results were identified, we performed Bonferroni pairwise comparison post-hoc tests. We assessed homogeneity of variance using Mauchly’s test of Sphericity, and wherever homogeneity of variance could not be assumed, Greenhouse-Geisser corrections were applied. All data were reported as means (M) and standard deviations (*SD*). For visual clarity, Figure error bars represented standard error mean (SEM). Partial eta-squared (η_p_^2^) and generalised eta-squared (η^2^*_G_*) were reported as the effect size for ANOVAs. Due to Cohen’s (1988) original partial eta-squared benchmarks of .01, .06 and .14 for small, medium and large effects, respectively, not being suitable for interpreting partial eta-squared for multi-factorial within-subjects designs (Lakens, 2013; Olejnik & Algina, 2003), we compared these benchmarks to generalized eta-squared which address considerations for which these benchmarks were based, as per Lakens (2013). Additionally, we reported Cohen’s *d* as the effect size for pairwise comparisons. In accordance with Cohen (1992), *d* values of 0.2, 0.5 and 0.8 indicated small, moderate, and large effects for pairwise comparisons, respectively. For all analyses, we set statistical significance at *p* < .05.^2^

## Results

### Manipulation Checks

#### Core Temperature

**Figure 2.**
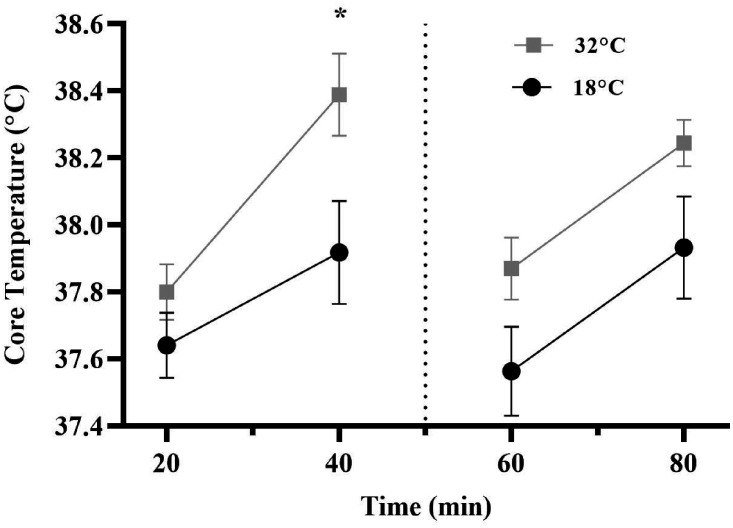
Core Temperature (˚C) as a Function of Time Between the Hot and Control Conditions. Note: * p <.05 between temperature conditions; the dashed line reflects half-time during the CISP.

We identified main effects for condition (*F*_1,11_ = 4.997, *p* = .047, η_p_^2^ = .312, η^2^*_G_* = .141), time (*F*_3,33_ = 24.735, *p* < .001, η_p_^2^ = .692, η^2^*_G_* = .214), and a condition × time interaction (*F*_3,33_ = 3.392, *p* = .029, η_p_^2^ = .236, η^2^*_G_* = .020) for core temperature. Bonferroni pairwise comparisons for the interaction revealed that, although no differences in core temperature were found in the first quarter of the CISP (*p* = .107, *d* = 0.42), core temperature was significantly higher in the hot (*M* = 38.40°C, *SD = *0.43) compared to the control condition (*M* = 37.92°C, *SD* = 0.53) (*p* = .015, *d* = 0.99) during the second quarter. Marginal differences were also observed in the third quarter of the CISP, whereby core temperature was higher in the hot (*M* = 37.87°C, *SD = *0.32) compared to the control condition (*M* = 37.56°C, *SD = *0.45) (*p* = .076, *d* = 0.79) (see Figure 2).

#### Thermal Sensation

There were main effects for condition (*F*_1,11_ = 13.426, *p* = .004, η_p_^2^ = .550, η^2^*_G_* = .372) and time (*F*_1.774,19.517_ = 17.513, *p < *.001, η_p_^2^ = .614, η^2^*_G_* = .210), but no time x condition interaction effect was observed (*F*_1.761, 19.373_ = 0.819, *p* = .441, η_p_^2^ = .069, η^2^*_G_* = .004) for thermal sensation. Participants’ reported higher thermal sensations in the hot condition (*M* = 6.08, *SD = *0.74) compared to the control condition (*M* = 4.88, *SD* = 0.69, *d* = 1.69). Additionally, reported thermal sensations were higher in the second (*M^diff^ = * + 0.83, *SD* = 0.66, *p* = .007, *d* = 1.64) and fourth (*M^diff^* = + 0.83, *SD* = 0.70, *p = *.011, *d* = 1.60) quarters compared to the first quarter. Also, thermal sensation was lower in the third quarter (*M^diff^* = − 0.75, *SD* = 0.54, *p* = .003, *d* = 1.33) compared to the second quarter, and higher in the fourth quarter (*M^diff^* = + 0.75, *SD* = 0.46, *p* = .001, *d* = 1.30) compared to the third quarter of the CISP (see Table 1).

**Table 1.** Descriptive Statistics (M ± SD) for HR, RPE, TSS and Affective Valence Across Quarters and Overall, Between Conditions.

		Time				
Variable	Condition	First	Second	Third	Fourth	Overall
HR	Control	146 ± 12 (∼76%)	155 ± 10 (∼81%)	149 ± 11 (∼78%)	155 ± 10 (∼81%)	151 ± 10 (∼79%)
	Hot	155 ± 10 (∼81%)	167 ± 12 (∼87%)	155 ± 10 (∼81%)	165 ± 9 (∼86%)	161 ± 10 (∼84%)
RPE	Control	12.25 ± 0.97	14.08 ± 1.40	13.17 ± 1.03	15.02 ± 1.25	13.63 ± 1.04
	Hot	13.19 ± 1.01	15.65 ± 1.50	14.17 ± 1.35	16.19 ± 1.40	14.80 ± 1.07
TSS	Control	4.52 ± 0.76	5.23 ± 0.83	4.54 ± 0.79	5.23 ± 0.97	4.88 ± 0.76
	Hot	5.56 ± 0.68	6.52 ± 0.80	5.71 ± 0.85	6.52 ± 0.79	6.08 ± 0.69
Valence	Control	2.67 ± 0.83	1.08 ± 1.43	1.90 ± 1.37	0.46 ± 1.83	1.53 ± 1.17
	Hot	2.08 ± 0.60	0.27 ± 1.42	1.18 ± 1.43	–0.15 ± 1.94	0.85 ± 1.11

*Note.* HR in presented in beats per minute with the average estimated % HRmax recorded in the VO^2^ Max tests reported in brackets.RPE was measured on a scale from 6 to 20, TSS from 0 to 8, and affective valence from –5 to +5.

### Performance Measures

#### Peak Power Output

**Figure 3.**
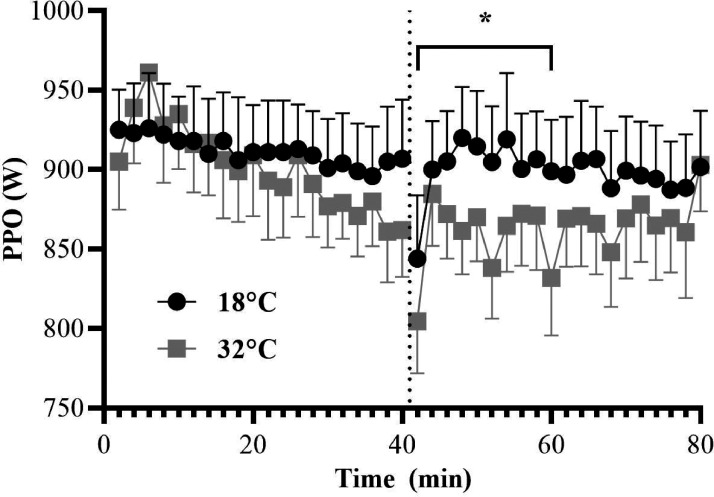
Peak Power Output (PPO) as a Function of Time (for quarters of the CISP) Between Temperature Conditions. Note: * p <.05 between temperature conditions; the dashed line reflects half-time during the CISP.

There was no main condition effect for PPO (*F*_1,11_ = 3.821, *p* = .076, η_p_^2^ = .258, η^2^*_G_* = .011), but there was a main effect for time(*F*_3,33_ = 9.060, *p* < .001, η_p_^2^ = .452, η^2^*_G_* = .021) and a significant condition × time interaction (*F*_1.445,4056.320_ = 5.537, *p* = .022, η_p_^2^ = .335, η^2^*_G_* = .010). Bonferroni pairwise comparisons observed that although no differences in mean PPO between temperature conditions were found in the first quarter of the CISP (*p* = .736, *d* = 0.03), mean PPO was lower in the hot (*M* = 861.31 W, *SD = *105.20) compared to the control (*M* = 900.68 W, *SD* = 114.83) condition in the third quarter of the CISP (*p* = .032, *d* = 0.36). Also, mean PPO was marginally lower in the hot compared to the control condition in the second (*M^diff^* = –25.03 W, *SD = *43.75, *p* = .073, *d* = 0.25) and fourth (*M^diff^* = –27.06 W, *SD = *45.76, *p* = .073, *d* = 0.25) quarters of the CISP (Figure 3).

#### Congruent Stroop

No differences were observed at rest between conditions for congruent Stroop RT performance (*F*_1,10_ = 1.677, *p* = .224, η_p_^2^ = .144, η^2^*_G_* = .036) or congruent Stroop accuracy (*F*_1,10_ = 1.000, *p* = .341, η_p_^2^ = .091, η^2^*_G_* < .001). For congruent RT during exercise, there was no main effect for condition (*F*_1,11_ = 1.326, *p* = .274, η_p_^2^ = .108, η^2^*_G_* = .008), however, a main effect for time (*F*_4,40_ = 3.037, *p* = .043, η_p_^2^ = .216, η^2^*_G_* = .016) and a significant condition × time interaction (*F*_3,33_ = 3.673, *p* = .022, η_p_^2^ = .250, η^2^*_G_* = .013) were found. Bonferroni pairwise comparisons identified that although no differences in RT were found in the first quarter (*p* = .185, *d* = 0.26), RT was quicker in the second quarter of the CISP in the hot condition (*M* = 561.99 ms, *SD* = 112.57) compared to the control condition (*M* = 617.80 ms, *SD* = 139.72) (*p* = .032, *d* = 0.44). However, no differences between conditions were found in the two quarters during the second half of the CISP (i.e., third and fourth quarters).

**Figure 4.**
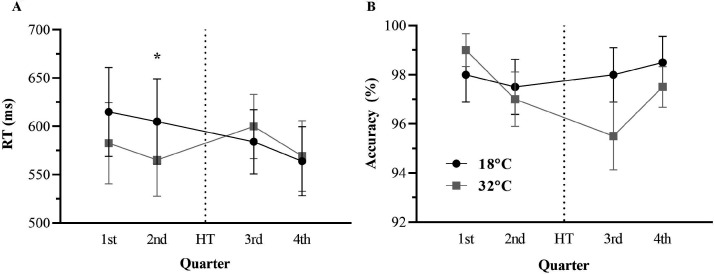
Congruent Stroop Reaction Time (RT; Panel A) and Accuracy (Panel B) as a Function of Time Between Temperature Conditions. Note: * p <.05 between temperature conditions; HT = half-time.

For Stroop congruent accuracy during exercise, there were no condition (*F*_1,11_ = 0.133, *p* = .723, η_p_^2^ = .012, η^2^*_G_* = .002), time (*F*_3,33_ = 1.713, *p* = .183, η_p_^2^ = .135, η^2^*_G_* = .034) nor condition × time interaction effects (*F*_3,33_ = 1.807, *p* = .165, η_p_^2^ = .141, η^2^*_G_* = .044) (see Figure 4).

#### Incongruent Stroop

No differences were observed at rest between temperature conditions for incongruent Stroop RT **(***F*_1.10_ = 0.085, *p* = .776, η_p_^2^ = .008, η^2^*_G_* = .005) or incongruent Stroop accuracy (*F*_1,10_ = 1.000, *p* = .341, η_p_^2^ = .091, η^2^*_G_* = .016). Similarly, during exercise trials, there was no significant effect for temperature condition (*F*_1,11_ = 0.056, *p* = .817, η_p_^2^ = .005, η^2^*_G_* < .001), time (*F*_3,33_ = 1.462, *p* = .243, η_p_^2^ = .117, η^2^*_G_* = .009) or condition × time interaction (*F*_3,33_ = 1.961, *p* = .139, η_p_^2^ = .151, η^2^*_G_* = .012) for incongruent Stroop RT.

**Figure 5.**
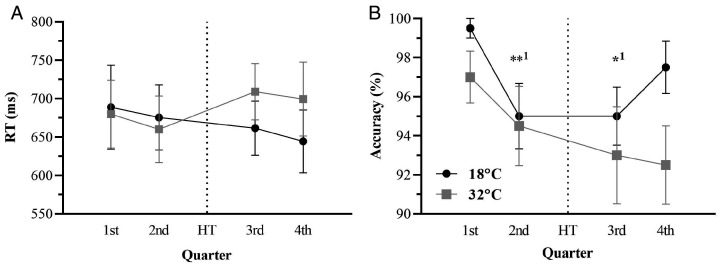
Incongruent Stroop RT (Panel A) and Accuracy (Panel B) as a Function of Time Between Temperature Conditions. Note: * p <.05, ** p <.01; 1=comparison with the first quarter; HT = half-time.

For incongruent Stroop accuracy, there was no condition (*F*_1,11_ = 2.723, *p* = .127, η_p_^2^ = .198, η^2^*_G_* = .042) nor condition × time interaction effect (*F*_3,33_ = 1.375, *p* = .268, η_p_^2^ = .111, η^2^*_G_* = .024). However, a main effect for time was identified (*F*_3,33_ = 6.136, *p* = .002, η_p_^2^ = .358, η^2^*_G_* = .107), whereby accuracy was significantly lower in the second (*M^diff^* = –3.75%, *SD = *3.11, *p* = .009, *d* = 1.14) and third (*M^diff^* = –4.58%, *SD = *4.24, *p* = .019, *d* = 1.15) quarters compared to the first quarter (*M* = 98.54%, *SD = *1.97) (Figure 5).

#### Vigilance

There were no differences at rest between temperature conditions for vigilance accuracy (*F*_1,10_ = 0.312, *p* = .588, η_p_^2^ = .030, η^2^*_G_* = .002) or vigilance RT (*F*_1,10_ = 1.459, *p* = .255, η_p_^2^ = .127, η^2^*_G_* = .022). Similarly, for vigilance accuracy during exercise, there were no temperature condition (*F*_1,11_ = 1.497, *p* = .247, η_p_^2^ = .120, η^2^*_G_* = .010), time (*F*_3,33_ = 0.391, *p* = .760, η_p_^2^ = .034, η^2^*_G_* = .003) nor condition × time interaction (*F*_3,33_ = 0.779, *p* = .514, η_p_^2^ = .066, η^2^*_G_* = .008) effects. Moreover, no main temperature condition (*F*_1,11_ = 0.004, *p* = .954, η_p_^2^ < .001, η^2^*_G_*< .001), time (*F*_3,33_ = 2.481, *p* = .078, η_p_^2^ = .184, η^2^*_G_* = .010) or interaction (*F*_3,33_ = 0.693, *p* = .563, η_p_^2^ = .059, η^2^*_G_* = .002) effects were found for vigilance RT.

#### HR

Regarding physiological measures, there were main HR effects for condition (*F*_1,11_ = 19.650, *p* = .001, η_p_^2^ = .641, η^2^*_G_* = .181), time (*F*_1.770,19.475_ = 37.898, *p* < .001, η_p_^2^ = .775, η^2^*_G_* = .170) and a condition × time interaction (*F*_1.701, 18.711_ = 4.706, *p* = .027, η_p_^2^ = .300, η^2^*_G_* = .011). Bonferroni pairwise comparisons revealed that although HR was significantly higher in the hot compared to control condition across all quarters, the differences were more salient and stronger in the second (*p* < .001, *d* = 1.18), third (*p* = .005, *d* = 1.63) and fourth (*p* < .001, *d* = 1.05) quarters of the CISP compared to the first (*p* = .014, *d* = 0.82) quarter (see Table 1).

#### Metanephrines

**Figure 6.**
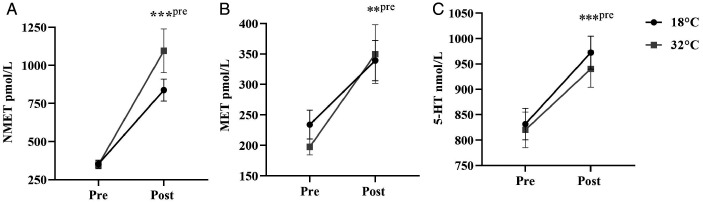
Normetanephrine (NMET) Concentration (Panel A), Metanephrine (MET) Concentration (Panel B) and Serotonin (5-HT) (Panel C) as a Function of Time Between Temperature Conditions. Note: ** p <.01; *** p <.001; pre = comparison with pre-CISP.

There was no main effect for temperature condition (*F*_1,10_ = 2.92, *p* = .083, η_p_^2^ = .270, η^2^*_G_* = .056), but there was a main effect for time (*F*_1,10_ = 48.464, *p* < .001, η_p_^2^ = .829, η^2^*_G_* = .583), and a marginal condition × time interaction (*F*_1,10_ = 4.116, *p* = .070, η_p_^2^ = .292, η^2^*_G_* = .060) for plasma indicator of norepinephrine (NMET) with large and moderate effect sizes, respectively. The interaction suggested that the increase in NMET from pre-CISP to post-CISP was more salient in the hot condition compared to the control condition. Specifically, no differences in NMET were found between temperature conditions pre-CISP (*p* = .874, *d* = 0.05), but a trend was found for NMET being higher in the hot condition compared to the control condition post-CISP (*p* = .072, *d* = 0.65) (see Figure 6A).

In terms of our plasma indicator of epinephrine (MET), no main effect for temperature condition (*F*_1,10_ = 0.039, *p* = .848, η_p_^2^ = .004, η^2^*_G_* < .001) was found. However, there was a significant main effect for time (*F*_1,10_ = 16.15, *p* = .002, η_p_^2^ = .618, η^2^*_G_* = .353), and a marginal condition × time interaction (*F*_1,10_  =  4.085, *p* = .071, η_p_^2^ = .290, η^2^*_G_* = .020). The marginal interaction indicated that the increase in MET from pre-CISP to post-CISP was greater in the hot condition (*M^diff^ = *+179.82 pmol/L, *SD* = 158.27, *p* = .004, *d* = 1.55) than in the control condition (*M^diff^ = *+122.64 pmol/L, SD = 102.55, *p* = .003, *d* = 1.25) (see Figure 6B).

#### Whole Blood 5-HT

For whole blood serotonin concentration (5-HT), no temperature condition (*F*_1,10_ = 0.070, *p* = .797, η_p_^2^ = .007, η^2^*_G_* < .001) or condition × time interaction (*F*_1,10_ = 0.714, *p* = .418, η_p_^2^ = .001, η^2^*_G_* < .001) effects were found. However, there was a main effect for time (*F*_1,10_ = 24.683, *p* < .001, η_p_^2^ = .712, η^2^*_G_* = .054), such that there was a significant concentration increase from pre-CISP to post-CISP (*M^diff = ^*+106.91 nmol/L, *SD = *74.56) (see Figure 6C).

#### RPE

There were main effects for RPE for temperature condition (*F*_1,11_ = 21.092, *p* = .001, η_p_^2^ = .657, η^2^*_G_* = .194) and time (*F*_3,33_ = 48.437, *p* < .001, η_p_^2^ = .815, η^2^*_G_* = .464), but no time × condition interaction effect was identified (*F*_3,33_ = 1.353, *p* = .274, η_p_^2^ = .110, η^2^*_G_* = .010). The main effects revealed that RPE was higher while participants exercised in the hot condition (*M = *14.80, *SD = *1.07) compared to the control condition (*M = *13.63, *SD = *1.04). Moreover, in terms of the time effect, significant differences were found between all quarters of the CISP (*p*s ≤ .019; *d*s = 0.60 to 2.65), and participants’ perceived exertion increased progressively from the first to the second half of the CISP (see Table 1).

#### Affective Valence

For affective valence, there was a main effect for temperature condition (F_1,11_ = 11.633, *p* = .006, η_p_^2^ = .514, η^2^*_G_* = .058) and time (*F*_3,33_ = 15.874, *p* < .001, η_p_^2^ = .591, η^2^*_G_* = .279), but no condition × time interaction effect (*F*_3,33_ = 0.158, *p* = .924, η_p_^2^ = .014, η^2^*_G_* = .001) was found. Participants reported feeling less pleasant in the hot condition (*M = *0.85, *SD = *1.11) than in the control condition (*M = *1.53, *SD = *1.17, *d* = 0.60). For the time effect, participants reported feeling lower pleasure across the first half of their exercise bouts, from the first to the second quarter (*M^diff^* = –1.70, *SD = *1.28, *p* = .004, *d* = 1.69), and again across the second half, from the third to the fourth quarter of the CISP (*M^diff^ = *−1.39, *SD* = 1.32, *p* = .022, *d* = 0.89). The largest decline in affective valence was observed from the first to the fourth quarter of the CISP (*M^diff^* = –2.22, *SD* = 1.59, *p* = .003, *d* = 1.67) (see Table 1).

### Fluid Intake and Urine Osmolality

A one-way repeated measures ANOVA revealed a significant difference in the total amount of water participants consumed between the two temperature conditions (*F*_1,11_ = 13.729, *p* = .003, η_p_^2^ = .555, η^2^*_G_* = .333), with more water consumed in the hot compared to the control condition (*M*^diff^ = 614.167 ml, *SD = *572.17).

In terms of urine osmolality, a 2 (condition) × 2 (time) ANOVA revealed no main effect for temperature condition (*F*_1,11_ = .867, *p* = .372, η_p_^2^ = .073, η^2^*_G_* = .030) nor time × condition interaction effect (*F*_1,11_ = .853, *p* = .376, η_p_^2^ = .072, η^2^*_G_* = .013). However, there was a significant time effect (F_1,11_ = 9.242, *p* = .011, η_p_^2^ = .457, η^2^*_G_* = .110), such that average urine osmolality increased from pre-CISP to post-CISP (*M*^diff^ = +170.08 mOsm/kg, *SD = *193.82) indicating that athletes were less hydrated post-CISP across both temperature conditions.

## Discussion

Prior to this study, researchers had yet to investigate the simultaneous effects of heat exposure on athletes’ physical performance, cognitive performance and catecholamines during prolonged intermittent exercise, reflective of match-play in team sports. We studied these variables to determine how cognitive performance is affected under different levels of thermal strain and to elucidate the biological mechanisms that may contribute to these effects, in order to inform future efforts to maintain optimal athletic performance in hot conditions.

Our primary findings concerning cognitive performance were that (a) incongruent Stroop accuracy deteriorated in response to prolonged intermittent sprint exercise regardless of environmental temperature conditions (albeit not in the fourth quarter of a match-like exercise bout), (b) congruent Stroop reaction time improved in the hot, compared to the control temperature condition in the first half of a match-like exercise bout, and (c) vigilance was unaffected by both prolonged intermittent sprint exercise and environmental temperature conditions. Physiologically, we found that (a) peak power output was significantly lower in the hot (32°C) condition compared to the control condition (18°C) in the 20-minutes immediately following half-time, and (b) normetanephrine and metanephrine concentration significantly increased from pre- to post-exercise, and there was a marginal effect toward greater increases in the hot, compared to the control, condition. These changes in peak power output and normetanephrine and metanephrine concentration may partially explain some the observed cognitive changes.

We found some evidence for incongruent Stroop accuracy deterioration in response to prolonged exercise in that these scores were lower in the second and third quarters, compared to the first quarter of participant exercise bouts. It is possible that this more complex Stroop task (i.e., incongruent Stroop) was reflective of higher cognitive functioning (i.e., interference control) that may be more sensitive to impairments during prolonged intermittent sprint exercise, even while Stroop RT and vigilance tasks were insensitive to these factors, as also found by Taylor et al. (2014). Others have also suggested that higher-order cognitive functioning (i.e., reflective of executive functioning) may be more sensitive to impairment when participants are more physically fatigued from exercise (e.g., Schmit & Brisswalter, 2018). This is because physical fatigue has seemed to result in an allocation of cerebral resources to regions responsible for the physical exercise demands, prioritizing those rather than frontal brain mediation of higher-order cognitive functions. Accordingly, the less complex cognitively demanding activities such as vigilance tasks, may be less sensitive to this prioritization process during exercise-induced fatigue.

The observed declines in Stroop incongruent accuracy over time during exercise may be explained by increases in metanephrine and normetanephrine that we identified from pre- to post-exercise. An extensive review by McMorris et al. (2011), concluded that an increase in catecholamine concentration in response to acute moderate or heavy exercise, results in increased speed of processing, but with a possible detrimental effect on cognitive accuracy due to increased neural ‘noise’. In the present study, we measured plasma metanephrine and normetanephrine as the more stable extra-neuronal metabolites of epinephrine and norepinephrine (Woods et al., 2017), and both were significantly increased from pre- to post-exercise across both temperature conditions. This explanation gains partial support from the deterioration in incongruent Stroop task accuracy we observed in the second and third quarters of exercise bouts.

Interestingly, congruent Stroop reaction time was also quicker in the hot condition compared to the control condition during the second quarter of exercise, while there were no condition differences in the first quarter, or indeed in any other quarters of the CISP. These findings partially align with research by Macleod et al. (2018) who identified elite hockey players’ visual search response speed to be faster post-exercise in a 33˚C compared to a 16˚C condition. These findings suggest a possible heightened response speed with increased core temperature, where core temperature in this study was only significantly higher in hot compared to the control condition in the second quarter of exercise. Additionally, it may be associated with the more accentuated catecholamine increase we observed, such that the marginal interaction reflected a greater increase in normetanephrine in the hot condition compared to the control condition. This biological change may have contributed to quicker speed of processing in the first half of the exercise bouts (McMorris et al., 2011). However, as no other differences were found between conditions across the latter quarters, a combination of factors may have contributed to this finding, including the participants’ lowered power output at the beginning of the second half of the CISP in the hot condition.

High concentrations of noradrenaline and adrenaline levels have been associated with over-arousal (McMorris et al., 2006), and affective and cognitive processes have been shown to be sensitive to arousal state (Berridge, 2008). In the hot condition in our study, physiological arousal (i.e. noradrenaline levels) appeared elevated, and participants felt less pleasant. Additionally, participants’ RPE and thermal sensations were also significantly higher in the hot condition (32°C), compared to the control condition (18°C), reflecting higher perceived strain in hotter conditions. Given previously reported relationships between neurotransmitter alteration and mood states (Davis & Bailey, 1997; Meeusen et al., 2006), willingness to maintain a high physical effort can be significantly influenced by central fatigue. These mechanisms may help explain why we found a lower mean peak power output in the third quarter of exercise in the hot condition compared to the control condition, although we found no differences during any other quarters. Our finding of lower than expected core temperatures in the hot condition may mean that reduced work output in hot conditions reduced the level of thermal strain experienced. This reduced thermal strain perhaps also explains why there were no significant temperature condition differences in incongruent Stroop accuracy scores and why there were no overall condition differences for incongruent Stroop accuracy in the fourth quarter compared to earlier quarters. Of note, a core temperature threshold of around 38.5˚C has previously been suggested as a criteria for when cognitive deficits begin to be identified (Schmit et al., 2017), where participants’ core temperature in this study reached heights of around 38.4˚C.

Previously, several studies investigating the effects of prolonged exercise on cognitive function within warm or hot conditions have used fixed-paced protocols (e.g., Taylor et al., 2014), or protocols in which a target work output (based on previous performance), and/or current work output is displayed to the participant (e.g., Coull et al., 2015). However, these methodologies impact the implementation of self-paced strategies that may better reflect real-world performance during match-play. Previous field-based research showed that soccer players could improve their technical performance (e.g. passing success rate) in ∼43°C compared to ∼21°C conditions, but they showed 41% less involvement in high-intensity running during the first half under the hotter condition (Mohr et al., 2012). Even though hyperthermia, normally known to contribute to a deteriorated cognitive performance, was associated in their study with a successful execution of technical skills, athletes seemed to have reduced their high-intensity work in the heat in order to compensate for the additional environmental stress (Mohr et al., 2012). This compensation may have aided the maintenance of technical skills when athletes were in possession of the ball in Mohr et al.’s (2012) study and when responding to cognitive tests throughout exercise in our research.

Although it is generally understood that dehydration in amounts as little as 2% can significantly impair physical and cognitive performance (Houssein et al., 2016), it is important to note that hydration status post-exercise was not different between conditions in our research, due to fluid intake having been significantly greater within the hot condition compared to the control condition. Our participants were able to consume fluid ad-libitum in alignment with previous research (Sunderland et al., 2015) and for reasons related to research ethics. This may also help explain why our participants showed a lack of significant cognitive deficits in the hotter, relative to the control, condition.

### Limitations and Future Research

Although this study offered novel insights, some limitations should also be acknowledged. The cognitive measures we employed within the present study were tasks that had been widely used in prior research and that varied in their level of complexity, but they were somewhat limited in ecological validity relative to the less predictable and more situation specific nature of match play decision-making within team sports. Despite extensive familiarization, the more generic cognitive tasks utilized were repetitive in nature and could be subject to learning effects (i.e., where participants re-familiarize with control panels). That said, the present study observed accuracy declines in the second and third quarters, and improved RT for congruent stimuli only in the second quarter of the hot condition. If there were learning effects, RT and accuracy improvements would be expected across conditions, therefore we are confident that the effects we observed resulted from the protocol’s demands. Furthermore, while laboratory research benefits from the control of extraneous variables, we were unable to completely replicate the competitive and motivational climates of in-game situations (e.g., crowd influence), which are likely to influence athletes physiological responses (e.g., arousal) as well as game-related cognitive variables (e.g., performance perceptions) (see Jones et al., 2007) that might influence the amount of effort invested even amidst heat exposure and fatigue.

Additionally, due to our use of a cycling based protocol to enable a variable work rate and simultaneously measure cognitive function, core temperatures were lower in our participants than those typically observed during team sports in the heat (∼39˚C) (Girard et al., 2015), and therefore more pronounced effects of condition could be expected with the implementation of a running protocol. Furthermore, we measured plasma metanephrines as markers of catecholamine secretion, rather than directly measuring cerebral functioning, due to practicality and a need to avoid the invasiveness of direct cortical measures. However, the relationship between blood plasma and cerebral plasma levels is still uncertain, and these data should be interpreted with care (Audhya et al., 2012; Goldstein, 2010). Finally, our small and all-male participant sample limits generalization of these results to other populations, especially women, particularly given the influence that menstrual cycle phase can have on physiological indices, such as core temperature and power output.

In the future, researchers should prioritize the use of less predictable and more sport-specific cognitive tasks to enhance the protocol’s ecological validity and reduce the influence of learning effects. Those seeking to understand how exercise parameters influence participants’ cognition must implement cognitive tests throughout exercise rather than only at pre- and immediately post-exercise in order to better replicate the simultaneous physical and cognitive demands of team sport. Additionally, where possible, researchers should expand the numbers and types of participants, particularly to better understand the relevance of women’s menstrual cycle fluctuations when considering study design and the time-frame of trials.

## Conclusions

Prolonged high-intensity intermittent exercise reduced accuracy on more complex cognitive tasks (incongruent Stroop), particularly in the second and third quarter of exercise, even though there were no changes in RT. Additionally, there was some evidence to suggest that prolonged high-intensity intermittent exercise with heat exposure (relative to control conditions) may facilitate RT on simple cognitive tasks (i.e., congruent Stroop), particularly through the first half of exercise bouts meant to mimic game play. More complex tasks requiring higher cognitive functioning may be more sensitive to impairments during prolonged exercise reflective of incremental fatigue in team sport (McMorris et al., 2011). Increases in metanephrine, and especially normetanephrine, from pre- to post-exercise support previous research suggesting that raised catecholamine levels may explain marginally faster simple cognitive responses in the fourth quarter compared to the third quarter, but less accurate complex cognitive performance during exercise (McMorris et al., 2011). We extended these findings by discovering that athletes employed lower physical work when exercising in 32°C compared to 18°C conditions, particularly early in the second half, perhaps reducing both their physiological strain and cognitive performance decrements, in the hotter condition. Team sport athletes seeking to optimize match performance in high temperature conditions should focus not only on reducing physiological strain, but also on sustaining a balance between both quick and accurate decision making for the entirety of match-play.

## Article Notes

**Author Biographies:** **Kate Donnan**, MSc, is completing a PhD in the psycho-physiological effects of heat stress on team sport performance at Leeds Beckett University, United Kingdom.

**Emily L. Williams**, PhD, is a Course Director in Sport, Exercise and Health Sciences and a Senior Lecturer in Sport and Exercise Physiology at Leeds Beckett University, United Kingdom.

**Nicholas Stanger**, PhD, is a Senior Lecturer at Leeds Beckett University. He received his PhD from the University of Birmingham in 2012.

## Notes

1 One participant was removed from normetanephrine and metanephrine analysis, and two participants 5-HT data could not be analysed, due to measurement error. Additionally, one participant’s pre-CISP normetanephrine result was winsorized, due to an extreme value from one trial that was outside of the normal range. For cognitive function at rest, *n* = 11 due to a technical fault occurring in testing one participant.

2 As our Power calculation was focused on main effects based on the relevant available literature and given that interactions require larger sample sizes to detect significant effects with the same magnitude (e.g., Fleiss, 1986; Leon & Heo, 2009), we placed greater consideration about the magnitude of effects when interpreting interactions (e.g., Hopkins, Batterham, Marshall, Hanin, 2009). For instance, to help avoid potential Type II error, we also identified marginal interaction effects, which we defined as *p* < .075 in the present study.
